# County-level variation and trajectories in ADHD medication dispensing among Norwegian children and adolescents, 2005-2022: a nationwide prescription register study

**DOI:** 10.3389/fpsyt.2026.1861616

**Published:** 2026-09-01

**Authors:** Espen Anker, Bothild Bendiksen, Gro Janne Wergeland, Per Hove Thomsen

**Affiliations:** 1 Klinikk Toppetasjen, Oslo, Norway; 2 Department of Clinical Medicine, Faculty of Medicine, University of Bergen, Bergen, Norway; 3 Department of Clinical Medicine, Aarhus University, Aarhus, Denmark; 4 Aarhus University Hospital, Aarhus, Denmark

**Keywords:** ADHD, geographical variation, Norway, prescription trends, regional variation, sex differences, stimulant medication

## Abstract

**Background:**

National trends in ADHD medication use in Norway have recently been described. Less is known about whether children and adolescents in different counties have followed similar or diverging medication-dispensing trajectories over time. We therefore examined county-level variation and county-level trajectories in ADHD medication dispensing among Norwegian children and adolescents from 2005 to 2022.

**Methods:**

We conducted a nationwide retrospective drug-utilisation study using aggregated data from the Norwegian Prescription Database and population denominators from Statistics Norway. The study included individuals aged 6–17 years who redeemed at least one prescription for an ADHD medication between 2005 and 2022. Annual dispensing prevalence was calculated by county, sex, and age group and expressed as users per 1,000 inhabitants. Analyses were stratified into children aged 6–11 years and adolescents aged 12–17 years. To quantify regional variation, we calculated the lowest and highest county rates, absolute rate differences, and high-to-low county rate ratios.

**Results:**

ADHD medication dispensing increased in all sex and age strata, but county-level trajectories differed over time. In 2022, dispensing prevalence among girls aged 6–11 years ranged from 4.6 per 1,000 inhabitants in Oslo to 15.1 per 1,000 in Møre og Romsdal, corresponding to a 3.25-fold difference. Among boys aged 6–11 years, rates ranged from 14.6 per 1,000 in Oslo to 39.9 per 1,000 in Nordland, corresponding to a 2.74-fold difference. Among adolescents aged 12–17 years, rates ranged from 15.3 per 1,000 in Agder to 35.2 per 1,000 in Trøndelag among girls, and from 25.1 per 1,000 in Oslo to 62.4 per 1,000 in Trøndelag among boys, corresponding to 2.30-fold and 2.49-fold differences, respectively.

**Conclusions:**

This study extends previous Norwegian register-based work by showing substantial county-level variation in ADHD medication dispensing among children and adolescents. National averages may conceal important regional differences in recognition, referral pathways, diagnostic practice, treatment thresholds, prescribing practice, or service organisation. Further studies linking prescription data with diagnostic, clinical, service-level, and socioeconomic information are needed to clarify the drivers and consequences of these regional differences.

## Introduction

Attention-Deficit/Hyperactivity Disorder (ADHD) is a neurodevelopmental condition characterised by persistent inattention, hyperactivity, and impulsivity that interfere with functioning and development (1). Global meta-analyses estimate that ADHD affects approximately 5–7% of school-aged children (2–4). ADHD often begins in childhood and may persist into adolescence and adulthood. Boys are more frequently diagnosed and treated than girls in childhood, although ADHD in girls may be less readily recognised, particularly when symptoms are predominantly inattentive or accompanied by internalising difficulties (5–7).

Over the last two decades, ADHD diagnoses and ADHD medication use have increased in several countries (8–10). Proposed explanations include increased clinical and public awareness, improved recognition of ADHD in girls and women, changes in diagnostic criteria, broader access to assessment, and greater acceptance of pharmacological treatment as part of multimodal care (11–13). Register-based studies have documented increasing use of both stimulant and non-stimulant ADHD medications among children and adolescents, alongside changes in medication availability and prescribing patterns (14–17).

Nordic prescription registers provide an opportunity to study ADHD medication dispensing at the population level. Recent Norwegian and Scandinavian studies have reported increasing ADHD medication use, including increasing treatment rates among females and changes in medication substance patterns over time (18, 19). In Norway, Hartz et al. recently described nationwide trends in ADHD medication use from 2006 to 2022 using data from the Norwegian Prescription Database (18). These national analyses provide an important reference point for understanding overall changes in ADHD medication use in Norway.

However, national averages may conceal regional differences. Geographical variation is an important dimension of ADHD diagnosis and treatment, particularly in countries with universal healthcare systems where large regional differences may raise questions about equity, access, referral pathways, diagnostic practice, treatment thresholds, and local prescribing cultures. International and Scandinavian studies have shown regional variation in ADHD diagnosis and medication use (20–22). In Norway, geographical variation has mainly been studied for ADHD diagnosis rather than medication dispensing. Previous Norwegian studies suggest that diagnosis rates vary more than measured symptom levels, and that clinician attitudes, local practice cultures, and service organisation may contribute to regional differences (23–25).

The present study was therefore designed to extend previous Norwegian register-based work by examining county-level variation and county-level trajectories in ADHD medication dispensing among Norwegian children and adolescents. The primary aim was to describe annual dispensing prevalence by county, sex, and age group among children aged 6–11 years and adolescents aged 12–17 years from 2005 to 2022. We also quantified the magnitude of regional variation by reporting absolute rate differences and high-to-low county rate ratios. Secondary aims were to describe national sex- and age-specific dispensing trends, first observed ADHD medication initiation in the Norwegian Prescription Database, and medication substance patterns over time.

## Methods

### Study design and setting

We conducted a nationwide retrospective drug-utilisation study of ADHD medication dispensing among children and adolescents in Norway from 1 January 2005 to 31 December 2022. The study used aggregated registry-derived data from the Norwegian Prescription Database (NorPD) (26, 27) and population denominators from Statistics Norway (28).

The primary focus was county-level variation and county-level trajectories in ADHD medication dispensing prevalence over time. National sex- and age-specific dispensing trends, first observed ADHD medication initiation in NorPD, and medication substance patterns were analysed as secondary outcomes.

### Data source and data processing

NorPD records all prescription medications dispensed from Norwegian pharmacies to individuals living outside institutions (26, 27). The database records redeemed prescriptions and therefore does not include prescriptions that were issued but not dispensed. Dispensing does not confirm ingestion, adherence, or continued treatment.

Data were obtained from NorPD via the Norwegian Medicines Agency and processed by Farmastat/Signum Life Science according to a predefined extraction specification. The specification included calendar year, sex, age group, county, encrypted person identifier, ATC code, and medication product information. Farmastat/Signum generated aggregated annual and county-level tabulations. Linkage across calendar years to define first observed ADHD medication initiation was performed using the encrypted person identifier within NorPD before aggregation. The authors did not have access to identifiable or individual-level NorPD data.

Medice Norway provided access to the statistical material but had no role in study design, data analysis, interpretation of findings, manuscript preparation, or the decision to submit the manuscript.

### Study population

The study population comprised all individuals aged 6–17 years who redeemed at least one prescription for an ADHD medication during the study period. Analyses were stratified by sex and by two age groups: children aged 6–11 years and adolescents aged 12–17 years. Age group was defined separately for each calendar year according to the age category available in the registry output.

County-level analyses were performed for each calendar year from 2005 to 2022. To allow comparison over time despite changes in Norwegian county structure, all county-level analyses were harmonised to the 11-county structure available for the whole study period. For years before county mergers, historical counties were aggregated to the corresponding later county groupings. Thus, Viken was constructed from Østfold, Akershus, and Buskerud; Innlandet from Hedmark and Oppland; Vestfold & Telemark from Vestfold and Telemark; Agder from Aust-Agder and Vest-Agder; Vestland from Hordaland and Sogn & Fjordane; Trøndelag from Sør-Trøndelag and Nord-Trøndelag; and Troms & Finnmark from Troms and Finnmark. Oslo, Rogaland, Møre & Romsdal, and Nordland were retained as separate counties throughout the study period.

### Medication exposure

ADHD medication exposure was defined as redemption of at least one medication with one of the following ATC codes: N06BA02 dexamfetamine, N06BA04 methylphenidate, N06BA07 modafinil, N06BA09 atomoxetine, N06BA12 lisdexamfetamine, and C02AC02 guanfacine. In the main analyses, medications were classified by active substance. Modafinil was included because the extraction was ATC-based, although it is not a standard first-line ADHD medication and is mainly used for other indications.

### Substance-level descriptive analyses

In the main prevalence and initiation analyses, individuals were counted once per calendar year regardless of the number of ADHD medications redeemed. Medication exposure was defined at the ATC/substance level.

**Figure 1:**
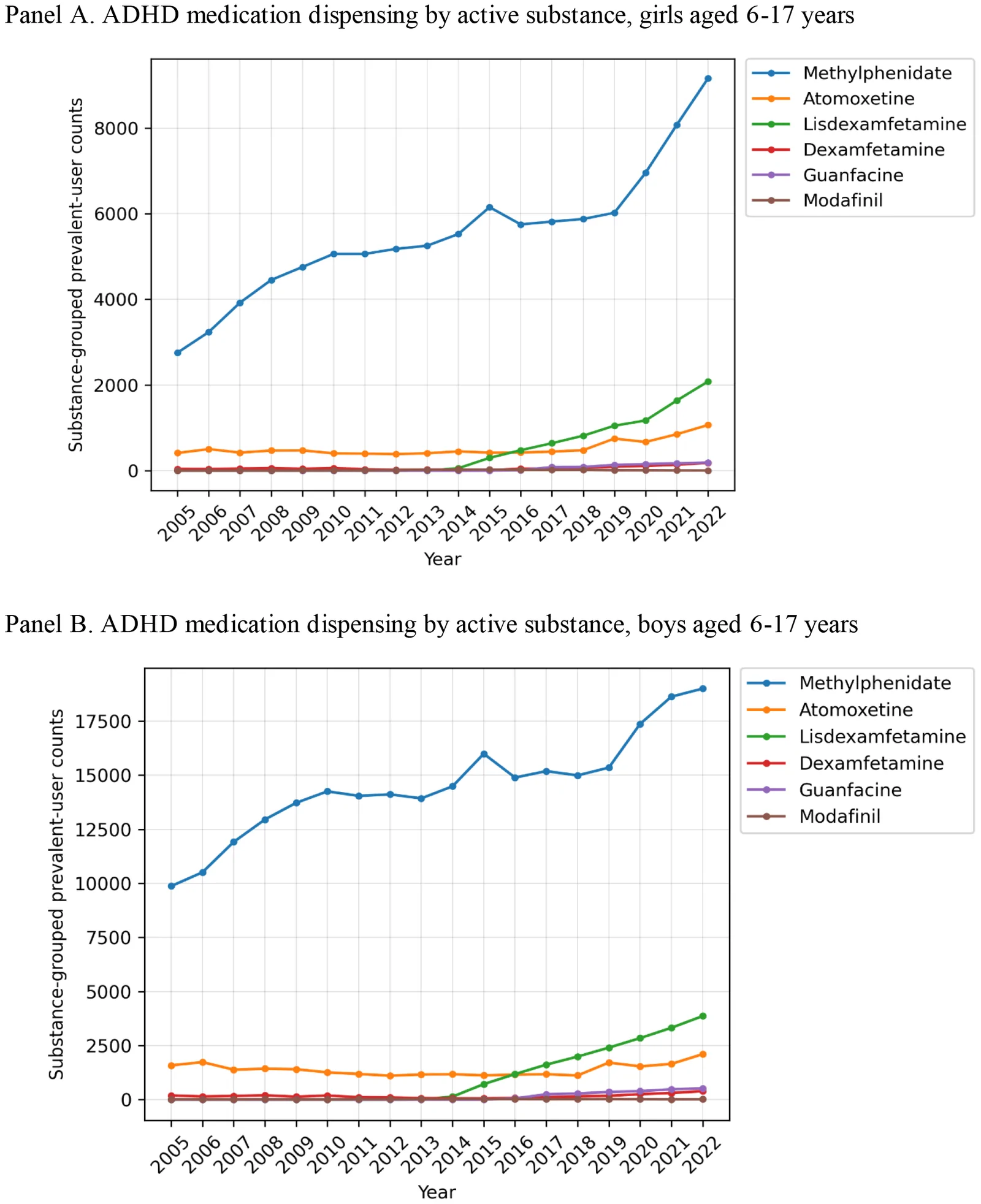
ADHD medication dispensing prevalence by active substance among users aged 6–17 years, Norway 2005-2022. Panel **(A)** shows girls and Panel **(B)** boys. Counts are based on aggregated product-level outputs classified into active-substance categories. Substance categories are non-mutually exclusive; the same individual may contribute to more than one active-substance category within a calendar year if more than one ADHD medication was dispensed. **(A)** ADHD medication dispensing by active substance, girls aged 6–17 years. **(B)** ADHD medication dispensing by active substance, boys aged 6–17 years.

Aggregated product-level outputs were available from the data provider and were used only to construct descriptive active-substance categories over time. For **Figure 1**, product-level outputs were aggregated into the following active-substance categories: methylphenidate, atomoxetine, lisdexamfetamine, dexamfetamine, guanfacine, and modafinil. These substance categories were non-mutually exclusive. Thus, an individual could contribute to more than one active-substance category within the same calendar year if more than one ADHD medication was dispensed. **Figure 1** should therefore be interpreted as describing dispensing patterns by active substance, not mutually exclusive patient-level treatment groups.

### Outcome definitions

For each calendar year, annual ADHD medication dispensing count was defined as the number of individuals in a given sex, age group, county, and calendar year who redeemed at least one ADHD medication.

Annual ADHD medication dispensing prevalence was defined as the annual dispensing count divided by the corresponding county-, sex-, age group-, and year-specific population denominator from Statistics Norway. Dispensing prevalence is reported as users per 1,000 inhabitants.

First observed ADHD medication initiation in NorPD was defined as the first observed redemption of any study ADHD medication during the NorPD observation period, with no previous redemption of any study ADHD medication recorded in NorPD from 2005 up to the year before the index year. This measure should not be interpreted as true lifetime treatment incidence, because ADHD medication use before 2005 and ADHD medication use outside Norway before immigration were not observable.

Individuals were counted once per calendar year in analyses of overall dispensing prevalence and first observed initiation, regardless of the number of dispensations or products redeemed. In product-level analyses, counts were non-mutually exclusive; the same individual could contribute to more than one product or substance category within the same year.

### Population denominators

Annual population denominators were obtained from Statistics Norway StatBank Table 07459 and were stratified by calendar year, sex, single-year age, and region (28). For national analyses, single-year age denominators were summed to match the study age groups 6–11 years and 12–17 years. For county-level analyses, county-specific single-year age denominators were summed into the same age groups and harmonised to the 11-county structure used in the manuscript.

For years before county mergers, denominators from historical counties were aggregated to match the corresponding manuscript county groupings. These denominator tables were then linked to the aggregated NorPD dispensing counts by calendar year, county, sex, and age group. County-specific dispensing prevalence was calculated as the number of ADHD medication users divided by the corresponding population denominator and expressed as users per 1,000 inhabitants.

### Back-calculation of selected initiation counts

For annual and county-level dispensing prevalence in 2022, the available outputs included original aggregated numerator counts. These counts were used together with Statistics Norway denominators to calculate rates and confidence intervals.

For first observed ADHD medication initiation, the available aggregated outputs included rates or percentages but did not include all underlying county-, sex-, and age-specific numerator counts in a deduplicated format. Where approximate counts were required for descriptive tabulation, estimated counts were derived by multiplying the reported rate or percentage by the relevant Statistics Norway denominator and rounding to the nearest whole number. These values are reported as estimated counts and were used for descriptive purposes only. Inferential analyses were not based on back-calculated counts.

### Statistical analysis

Descriptive statistics were used to summarise annual ADHD medication dispensing prevalence by county, sex, age group, and calendar year. The main regional analyses described county-level trajectories from 2005 to 2022 and county-level differences in 2022. To summarise regional variation over time, we calculated, for each calendar year and sex-age stratum, the lowest county rate, the median county rate, and the highest county rate. We also calculated county-specific rate ratios comparing 2022 with 2005.

To quantify the magnitude of regional variation, we calculated, for each sex and age stratum in 2022, the lowest county rate, the highest county rate, the absolute rate difference between the highest and lowest county, and the high-to-low county rate ratio. These measures were used as descriptive indicators of geographical variation and were interpreted alongside county-specific prevalence estimates and confidence intervals.

Where original numerator counts were available, 95% confidence intervals for proportions were calculated using binomial methods. Rate ratios with 95% confidence intervals were calculated on the log scale. County differences in 2022 were examined by comparing county-specific proportions of individuals redeeming ADHD medication. Heterogeneity across counties was assessed using binomial regression models with the number of users as the outcome and the relevant population denominator as the binomial denominator.

Likelihood-ratio tests were used to assess county effects and sex-by-county interaction. Where multiple county-level comparisons were reported, Bonferroni adjustment was applied. A two-sided p value <0.05 was considered statistically significant.

Temporal trends were primarily analysed descriptively using annual counts, annual population-based rates, and graphical presentation. Regression-based temporal trend models were not used as the main analysis because the objective was descriptive and because the observed time trends were non-linear, particularly in the latter part of the observation period.

Analyses were performed using IBM SPSS Statistics version 24 and Microsoft Excel.

### Disclosure control and quality control

To comply with data-provider disclosure rules, cell counts below five in the supplied aggregated outputs were disclosure-protected. In the available tables, such cells were represented as 2. These values were retained for descriptive calculations but should be interpreted as approximations rather than exact cell counts.

Quality-control procedures included checking consistency between aggregated annual totals and stratified counts, recalculating rates from supplied counts and Statistics Norway denominators, checking that figures matched the underlying tables, and verifying that individuals were counted once per year in the overall analyses. Product-level analyses were checked separately because product counts were non-mutually exclusive. Denominator totals were checked by summing single-year age groups and comparing the results with national population totals for the corresponding sex and age group.

### Ethics and approvals

The study was based on anonymised registry-derived data. Ethical approval was granted by the Regional Committee for Medical and Health Research Ethics (REK #344237). Individual informed consent was waived under Norwegian regulations for anonymised registry-based research.

### Data availability

The raw NorPD data used for this study are subject to Norwegian legal and ethical restrictions and cannot be made publicly available by the authors. Aggregated derived data underlying the figures and tables may be available from the corresponding author upon reasonable request, subject to permissions from the relevant data providers and applicable regulations.

The **Supplementary Material** includes county-, sex-, age group-, and year-specific dispensing prevalence rates from 2005 to 2022, the population denominators used for rate calculation, and supplementary county-specific trajectory figures.

### Role of AI-assisted language editing

ChatGPT was used for language editing and manuscript structuring. No AI tool was used for study design, data extraction, statistical analysis, or interpretation of the findings.

## Results

### County-level variation and magnitude of regional differences

**Figure 2:**
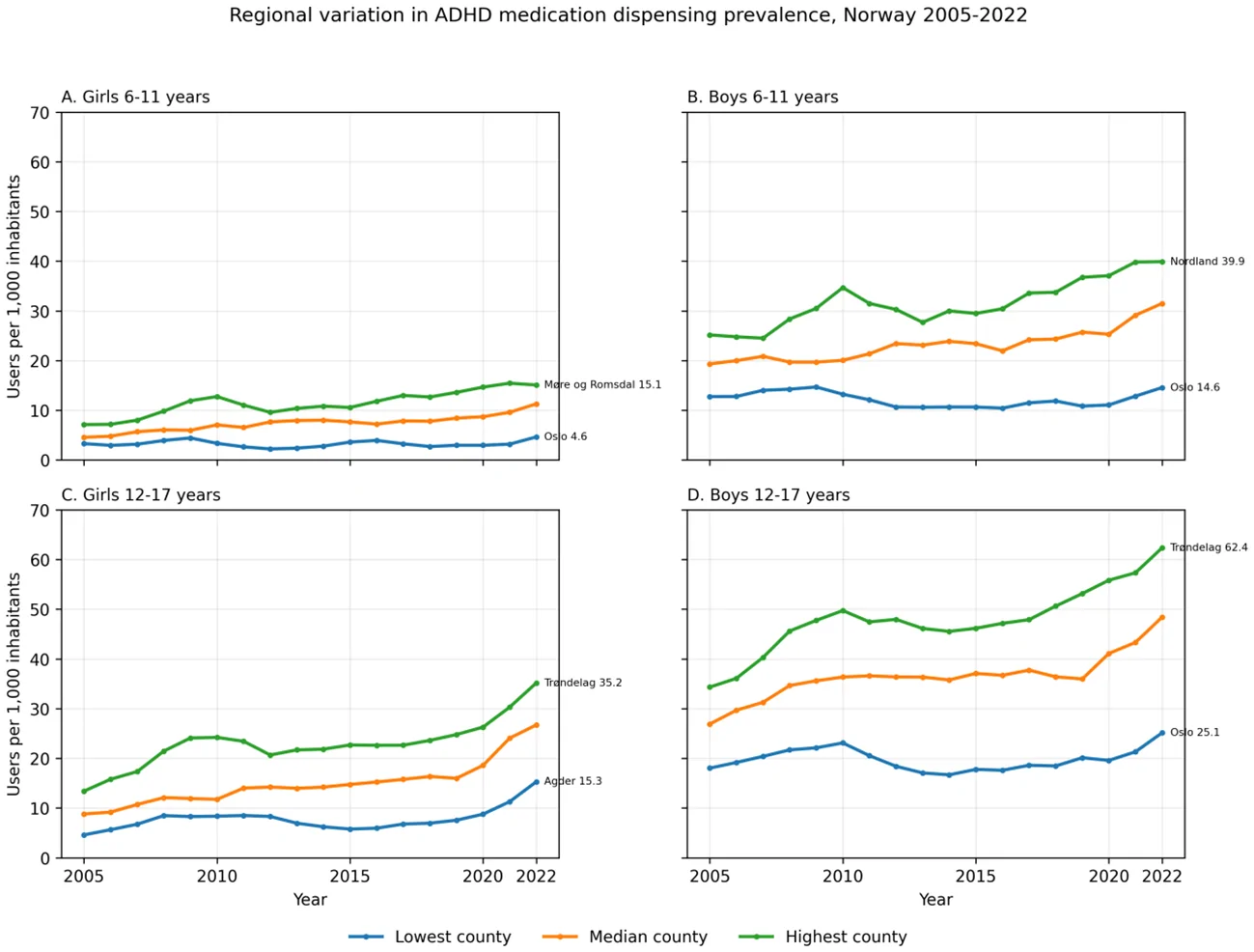
Regional variation in ADHD medication dispensing prevalence over time among Norwegian children and adolescents, Norway 2005-2022. **(A)** shows girls aged 6–11 years, **(B)** boys aged 6–11 years, **(C)** girls aged 12–17 years, and **(D)** boys aged 12–17 years. In each panel, the lowest county rate, the median county rate, and the highest county rate are shown for each calendar year. Rates are expressed as users per 1,000 inhabitants and were calculated as the number of individuals redeeming at least one ADHD medication prescription during the calendar year divided by the corresponding county-, sex-, age-, and year-specific population denominator from Statistics Norway.

County-level variation in ADHD medication dispensing prevalence was observed throughout the study period in all sex and age strata (**Figure 2**; **Supplementary Figures 1–S4**). The regional trend figures showed that counties differed not only in their 2022 dispensing rates, but also in their long-term trajectories from 2005 to 2022.

In 2022, dispensing prevalence among girls aged 6–11 years ranged from 4.6 per 1,000 inhabitants in Oslo to 15.1 per 1,000 in Møre og Romsdal, corresponding to a 3.25-fold difference. Among boys aged 6–11 years, rates ranged from 14.6 per 1,000 in Oslo to 39.9 per 1,000 in Nordland, corresponding to a 2.74-fold difference.

Among adolescents aged 12–17 years, county-level dispensing prevalence in 2022 ranged from 15.3 per 1,000 in Agder to 35.2 per 1,000 in Trøndelag among girls, and from 25.1 per 1,000 in Oslo to 62.4 per 1,000 in Trøndelag among boys, corresponding to 2.30-fold and 2.49-fold differences, respectively.

To quantify the magnitude of county-level variation, we calculated absolute rate differences and high-to-low county rate ratios for each sex and age stratum in 2022 (**Table 1**). The largest relative county difference was observed among girls aged 6–11 years, where dispensing prevalence was 3.25 times higher in Møre og Romsdal than in Oslo. The largest absolute county difference was observed among boys aged 12–17 years, with a difference of 37.3 users per 1,000 inhabitants between Trøndelag and Oslo.

**Table 1:** Magnitude of county-level variation in ADHD medication dispensing prevalence, Norway 2022.

Sex and age group	Lowest county rate	Highest county rate	Absolute rate difference	High-to-low county rate ratio
Girls 6–11 years	Oslo: 4.6	Møre og Romsdal: 15.1	10.5	3.25
Boys 6–11 years	Oslo: 14.6	Nordland: 39.9	25.3	2.74
Girls 12–17 years	Agder: 15.3	Trøndelag: 35.2	19.9	2.30
Boys 12–17 years	Oslo: 25.1	Trøndelag: 62.4	37.3	2.49

Rates are users per 1,000 inhabitants. Absolute rate difference denotes highest minus lowest county rate. High-to-low county rate ratio denotes highest county rate divided by lowest county rate. County-specific rates are shown in **Table 3**.

**Figure 3:**
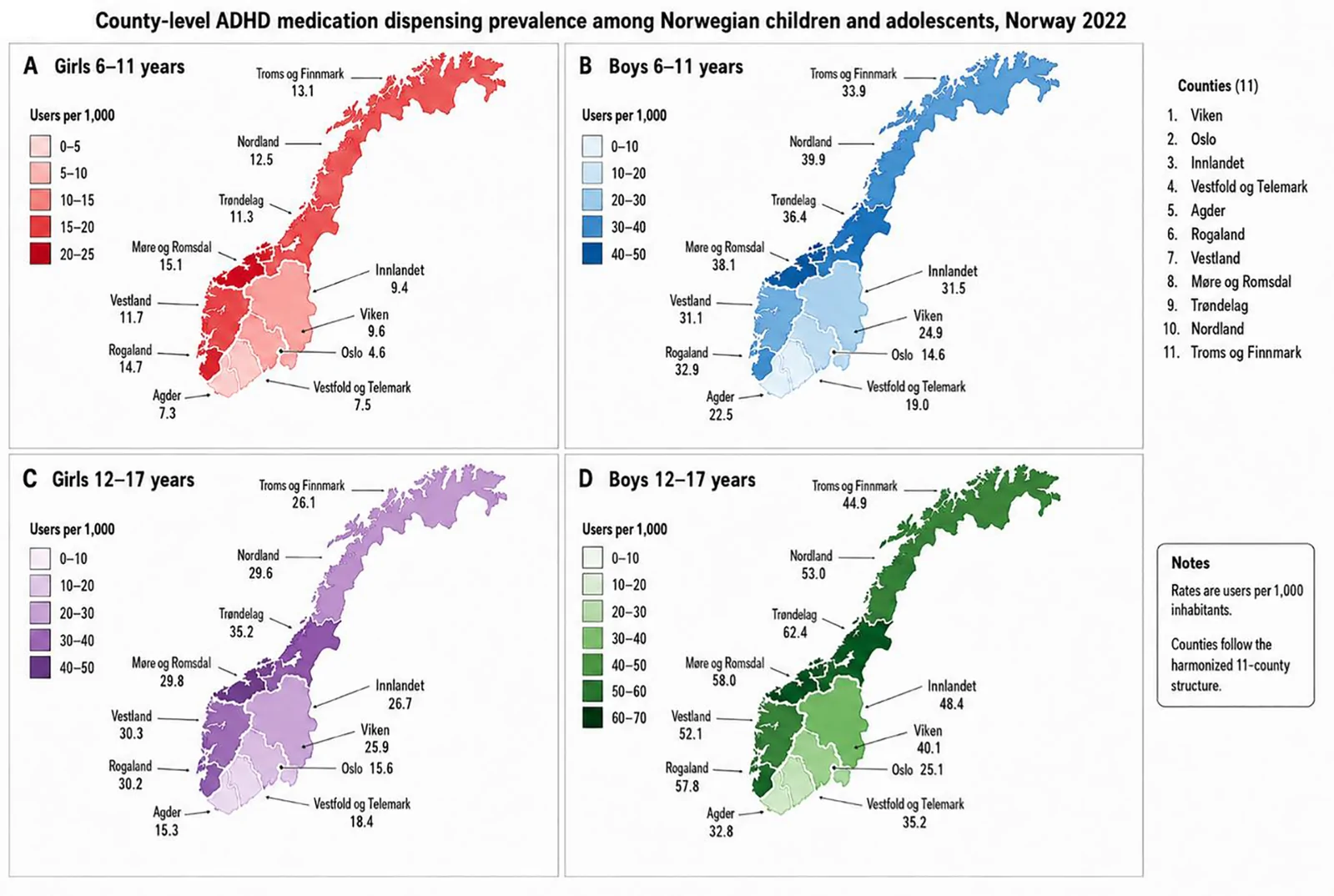
County-level ADHD medication dispensing prevalence among Norwegian children and adolescents, Norway 2022. Panel **(A)** shows girls aged 6–11 years, Panel **(B)** boys aged 6–11 years, Panel **(C)** girls aged 12–17 years, and Panel **(D)** boys aged 12–17 years. Rates are expressed as users per 1,000 inhabitants and were calculated as the number of individuals redeeming at least one ADHD medication prescription during 2022 divided by the corresponding county-, sex-, and age-group-specific population denominator from Statistics Norway. County groupings correspond to the harmonised 11-county structure used in the manuscript.

The geographical distribution of county-level dispensing prevalence in 2022 is shown in **Figure 3**. Oslo was consistently among the counties with the lowest rates across sex and age strata, whereas higher adolescent rates were observed in several western, central, and northern counties. Among adolescents, Trøndelag had the highest dispensing prevalence in both girls and boys. These geographical patterns illustrate that the national increase in ADHD medication dispensing was accompanied by marked regional heterogeneity.

County differences in dispensing prevalence in 2022 were statistically significant in all sex and age strata. In binomial regression models, county remained associated with dispensing prevalence after adjustment for sex within both age groups. There was also evidence of sex-by-county interaction, indicating that the magnitude of the sex difference varied across counties: p = 0.020 among children aged 6–11 years and p = 0.003 among adolescents aged 12–17 years.

### Regional trajectories over time

The longitudinal min-median-max plots showed that county differences were not limited to a single year (**Figure 2**). Among girls aged 12–17 years, the highest county rate increased from 13.4 per 1,000 in 2005 to 35.2 per 1,000 in 2022, while the median county rate increased from 8.8 to 26.7 per 1,000 and the lowest county rate increased from 4.6 to 15.3 per 1,000.

The supplementary county-specific trajectory figures show that some counties remained among the lower-rate or higher-rate counties across much of the observation period, whereas others showed steeper increases in the latter years. These findings indicate that regional differences reflected both differences in level and differences in trajectory over time.

### National sex- and age-specific dispensing trends

**Figure 4:**
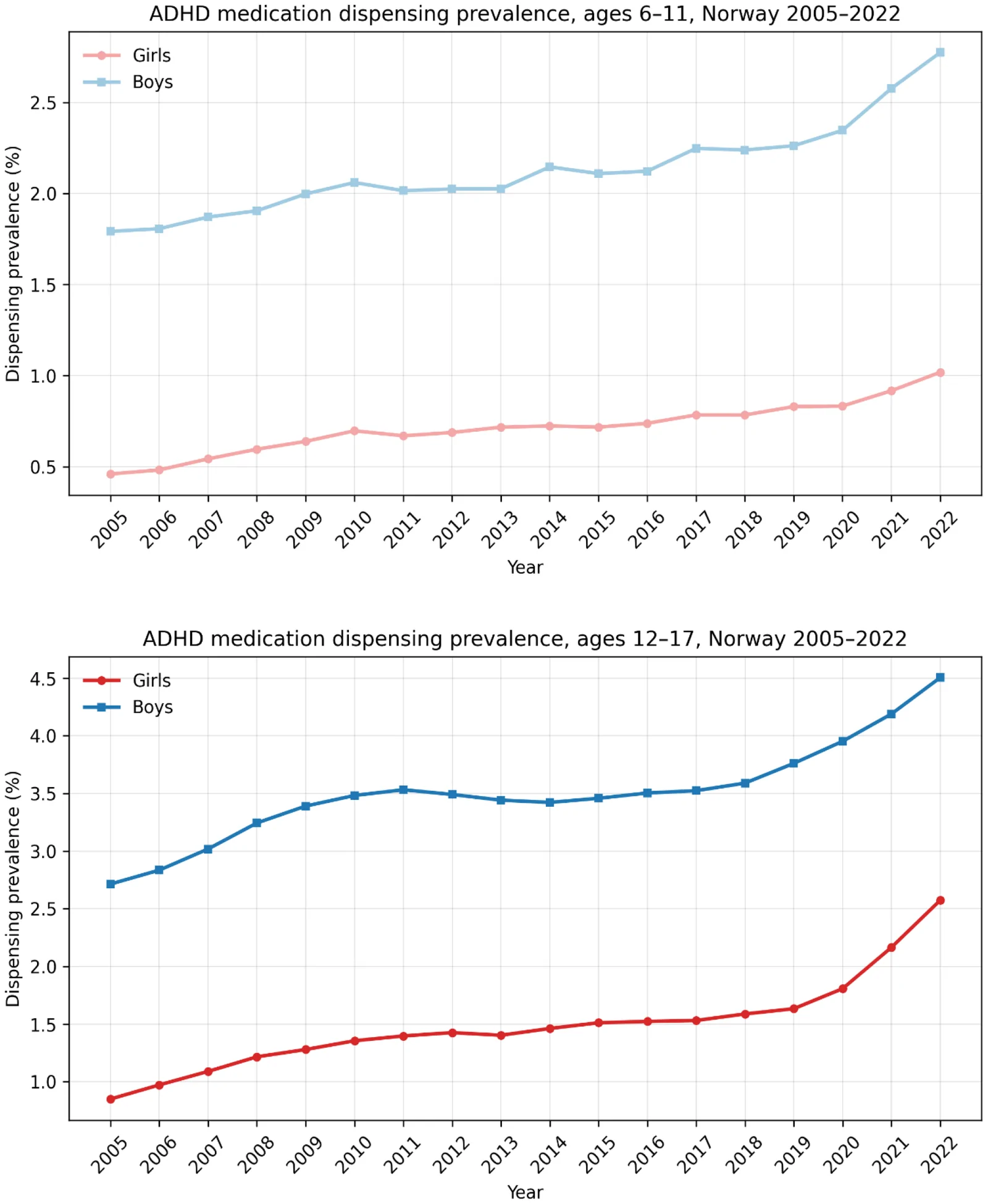
Annual ADHD medication dispensing prevalence among Norwegian children and adolescents, 2005-2022. **(A)** shows children aged 6–11 years and Panel **(B)** shows adolescents aged 12–17 years. Prevalence was calculated as the number of individuals redeeming at least one ADHD medication prescription during the calendar year divided by the corresponding sex-, age-, and year-specific population denominator from Statistics Norway.

As secondary context, annual ADHD medication dispensing prevalence increased in all sex and age strata between 2005 and 2022 (**Table 2**; **Figure 4**). County-specific dispensing prevalence estimates for 2022 are presented in **Table 3**. In 2005, 2,352 girls aged 6–17 years redeemed at least one ADHD medication prescription, corresponding to 0.65% of the female population in this age range. Among boys, 8,536 redeemed at least one ADHD medication prescription, corresponding to 2.25%. In 2022, 6,765 girls aged 6–17 years redeemed at least one ADHD medication prescription, corresponding to 1.81%, while 14,395 boys did so, corresponding to 3.65%.

**Table 2:** Dispensing prevalence are individuals aged 6–17 years redeeming ≥1 ADHD medication during the calendar year. Population denominators are from Statistics Norway StatBank Table 07459.

Year	Girls 6–11 n/N	Girls 6–11% (95% CI)	Boys 6–11 n/N	Boys 6–11% (95% CI)	Girls 12–17 n/N	Girls 12–17% (95% CI)	Boys 12–17 n/N	Boys 12–17% (95% CI)
2005	829/180 207	0.46 (0.43–0.49)	3 404/189 997	1.79 (1.73–1.85)	1 523/179 128	0.85 (0.81–0.89)	5 132/189 075	2.71 (2.64–2.79)
2006	869/180 235	0.48 (0.45–0.52)	3 437/190 268	1.81 (1.75–1.87)	1 772/182 136	0.97 (0.93–1.02)	5 451/192 274	2.84 (2.76–2.91)
2007	978/180 132	0.54 (0.51–0.58)	3 553/189 958	1.87 (1.81–1.93)	2 003/183 793	1.09 (1.04–1.14)	5 858/194 071	3.02 (2.94–3.10)
2008	1 068/179 218	0.60 (0.56–0.63)	3 590/188 482	1.90 (1.84–1.97)	2 249/184 903	1.22 (1.17–1.27)	6 342/195 512	3.24 (3.17–3.32)
2009	1 136/177 788	0.64 (0.60–0.68)	3 717/186 097	2.00 (1.93–2.06)	2 375/185 598	1.28 (1.23–1.33)	6 664/196 547	3.39 (3.31–3.47)
2010	1 235/177 221	0.70 (0.66–0.74)	3 815/185 204	2.06 (2.00–2.13)	2 519/185 847	1.36 (1.30–1.41)	6 870/197 287	3.48 (3.40–3.56)
2011	1 188/177 381	0.67 (0.63–0.71)	3 735/185 354	2.02 (1.95–2.08)	2 599/186 026	1.40 (1.34–1.45)	6 959/197 037	3.53 (3.45–3.61)
2012	1 219/177 255	0.69 (0.65–0.73)	3 742/184 823	2.02 (1.96–2.09)	2 657/186 330	1.43 (1.37–1.48)	6 890/197 379	3.49 (3.41–3.57)
2013	1 276/177 957	0.72 (0.68–0.76)	3 759/185 572	2.03 (1.96–2.09)	2 621/186 802	1.40 (1.35–1.46)	6 799/197 552	3.44 (3.36–3.52)
2014	1 300/179 649	0.72 (0.69–0.76)	4 027/187 677	2.15 (2.08–2.21)	2 720/186 060	1.46 (1.41–1.52)	6 708/196 031	3.42 (3.34–3.50)
2015	1 308/182 354	0.72 (0.68–0.76)	4 034/191 270	2.11 (2.05–2.17)	2 786/184 321	1.51 (1.46–1.57)	6 698/193 705	3.46 (3.38–3.54)
2016	1 369/185 430	0.74 (0.70–0.78)	4 135/194 848	2.12 (2.06–2.19)	2 801/183 885	1.52 (1.47–1.58)	6 766/193 118	3.50 (3.42–3.59)
2017	1 473/187 858	0.78 (0.75–0.83)	4 440/197 548	2.25 (2.18–2.31)	2 821/184 219	1.53 (1.48–1.59)	6 859/194 654	3.52 (3.44–3.61)
2018	1 483/189 150	0.78 (0.75–0.82)	4 463/199 372	2.24 (2.17–2.30)	2 923/184 059	1.59 (1.53–1.65)	6 957/193 825	3.59 (3.51–3.67)
2019	1 569/189 014	0.83 (0.79–0.87)	4 511/199 451	2.26 (2.20–2.33)	3 011/184 343	1.63 (1.58–1.69)	7 279/193 561	3.76 (3.68–3.85)
2020	1 570/188 510	0.83 (0.79–0.87)	4 656/198 430	2.35 (2.28–2.41)	3 355/185 642	1.81 (1.75–1.87)	7 701/194 867	3.95 (3.87–4.04)
2021	1 709/186 390	0.92 (0.87–0.96)	5 051/196 061	2.58 (2.51–2.65)	4 065/187 777	2.16 (2.10–2.23)	8 281/197 698	4.19 (4.10–4.28)
2022	1 871/183 754	1.02 (0.97–1.07)	5 363/193 373	2.77 (2.70–2.85)	4 894/190 175	2.57 (2.50–2.65)	9 032/200 528	4.50 (4.41–4.60)

Confidence intervals are Wilson binomial confidence intervals. Cell counts below five in supplied aggregated outputs were represented as 2.

Among children aged 6–11 years, dispensing prevalence increased from 0.46% in girls and 1.79% in boys in 2005 to 1.02% in girls and 2.77% in boys in 2022. Boys had higher dispensing prevalence than girls throughout the study period, although the girl-to-boy prevalence ratio increased from 0.26 in 2005 to 0.37 in 2022.

Among adolescents aged 12–17 years, dispensing prevalence increased from 0.85% in girls and 2.71% in boys in 2005 to 2.57% in girls and 4.50% in boys in 2022. The increase was particularly pronounced among adolescent girls in the latter part of the study period. From 2019 to 2022, the number of treated adolescent girls increased from 3,011 to 4,894, while dispensing prevalence increased from 1.63% to 2.57%. During the same period, the number of treated adolescent boys increased from 7,279 to 9,032, while dispensing prevalence increased from 3.76% to 4.50%. The sex gap therefore narrowed among adolescents, with the girl-to-boy prevalence ratio increasing from 0.31 in 2005 to 0.57 in 2022.

### First observed ADHD medication initiation in NorPD

**Figure 5:**
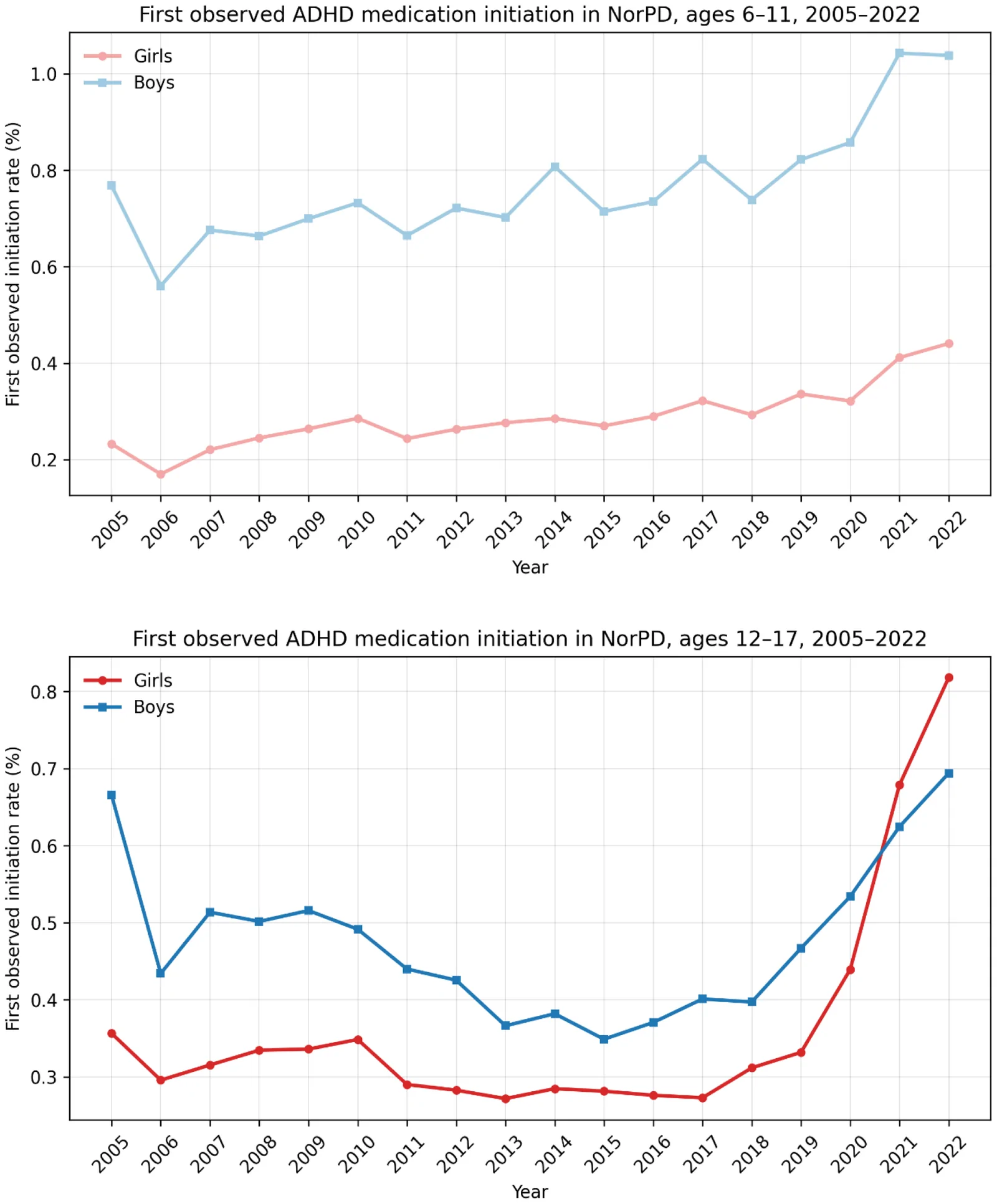
First observed ADHD medication initiation in the Norwegian Prescription Database among children and adolescents, 2005-2022. Panel **(A)** shows children aged 6–11 years and Panel **(B)** shows adolescents aged 12–17 years. Initiation rates were calculated using the available aggregated initiation outputs and the corresponding sex-, age-, and year-specific population denominators from Statistics Norway. These rates should be interpreted as first observed initiation within the NorPD observation period, not as true lifetime treatment incidence.

**Figure 5** shows annual first observed ADHD medication initiation in the Norwegian Prescription Database by sex and age group. Among children aged 6–11 years, initiation rates were higher among boys than girls throughout the study period, and changes over time were more modest than among adolescents.

**Table 3:** ADHD medication dispensing prevalence by county, sex and age group, Norway 2022.

County	Girls 6–11 n/N	Girls 6–11 per 1,000 (95% CI)	Boys 6–11 n/N	Boys 6–11 per 1,000 (95% CI)
Viken	435/45 256	9.6 (8.7–10.6)	1 182/47 429	24.9 (23.5–26.4)
Oslo	98/21 120	4.6 (3.8–5.7)	325/22 319	14.6 (13.0–16.2)
Innlandet	104/11 115	9.4 (7.7–11.3)	371/11 781	31.5 (28.4–34.8)
Vestfold og Telemark	103/13 773	7.5 (6.1–9.1)	278/14 629	19.0 (16.9–21.3)
Agder	82/11 183	7.3 (5.8–9.1)	267/11 884	22.5 (19.9–25.3)
Rogaland	279/19 038	14.7 (13.0–16.5)	651/19 765	32.9 (30.5–35.5)
Vestland	260/22 181	11.7 (10.3–13.2)	737/23 660	31.1 (29.0–33.4)
Møre og Romsdal	136/9 017	15.1 (12.7–17.8)	358/9 387	38.1 (34.4–42.2)
Trøndelag	179/15 853	11.3 (9.7–13.1)	609/16 742	36.4 (33.6–39.3)
Nordland	96/7 657	12.5 (10.2–15.3)	320/8 021	39.9 (35.7–44.4)
Troms og Finnmark	99/7 561	13.1 (10.7–15.9)	263/7 756	33.9 (30.0–38.2)
	Girls 12–17 n/N	Girls 12–17per 1,000 (95% CI)	Boys 12–17 n/N	Boys 12–17 per 1,000 (95% CI)
Viken	1 222/47 178	25.9 (24.5–27.4)	2 008/50 029	40.1 (38.4–41.9)
Oslo	318/20 325	15.6 (14.0–17.4)	527/20 957	25.1 (23.1–27.4)
Innlandet	325/12 161	26.7 (23.9–29.7)	631/13 035	48.4 (44.8–52.2)
Vestfold og Telemark	271/14 694	18.4 (16.3–20.8)	558/15 854	35.2 (32.4–38.2)
Agder	182/11 898	15.3 (13.2–17.7)	402/12 261	32.8 (29.7–36.1)
Rogaland	566/18 762	30.2 (27.8–32.7)	1 134/19 631	57.8 (54.5–61.1)
Vestland	698/23 046	30.3 (28.1–32.6)	1 252/24 025	52.1 (49.3–55.0)
Møre og Romsdal	290/9 732	29.8 (26.5–33.4)	590/10 172	58.0 (53.5–62.7)
Trøndelag	571/16 234	35.2 (32.4–38.1)	1 074/17 225	62.4 (58.8–66.1)
Nordland	238/8 049	29.6 (26.0–33.5)	460/8 673	53.0 (48.4–58.0)
Troms og Finnmark	211/8 096	26.1 (22.7–29.8)	389/8 666	44.9 (40.6–49.5)

Rates are users per 1,000 inhabitants; 95% confidence intervals are binomial confidence intervals.

n/N denotes dispensing prevalence divided by the county-, sex-, and age-group-specific population denominator. Rates are users per 1,000 inhabitants. County aggregation: Viken = Østfold + Akershus + Buskerud; Vestfold og Telemark = Vestfold + Telemark; Troms og Finnmark = Troms + Finnmark.

Among adolescents aged 12–17 years, initiation increased more clearly in the latter part of the observation period, particularly among girls. From 2019 onward, first observed initiation among adolescent girls increased more steeply than among adolescent boys, and by the final study years girls had higher initiation rates than boys. These results should be interpreted as first observed ADHD medication initiation within the NorPD observation period, not as true lifetime treatment incidence.

### Medication substance patterns among ADHD medication users

**Figure 1** shows ADHD medication dispensing prevalence by active substance among users aged 6–17 years. Methylphenidate-based medications dominated throughout the study period in both girls and boys. Over time, the range of dispensed ADHD medications expanded. Atomoxetine and dexamfetamine were present throughout most of the observation period, while lisdexamfetamine and guanfacine contributed to increased medication diversity after becoming available in Norwegian clinical practice.

These analyses were based on aggregated product-level outputs classified into active-substance categories. The categories are non-mutually exclusive; the same individual could contribute to more than one active-substance category within a calendar year if more than one ADHD medication was dispensed. These findings should therefore be interpreted as descriptive dispensing patterns by active substance, not as mutually exclusive treatment groups.

## Discussion

The main finding of this nationwide prescription register study was that ADHD medication dispensing among Norwegian children and adolescents showed substantial county-level variation from 2005 to 2022. Regional differences were observed in both age groups and in both sexes, and they were not limited to the final study year. In 2022, the highest county rates were approximately 2.3 to 3.3 times the lowest county rates across the four sex and age strata. The largest relative difference was observed among girls aged 6–11 years, whereas the largest absolute difference was observed among boys aged 12–17 years. These findings indicate that national averages conceal marked regional heterogeneity in ADHD medication dispensing among Norwegian children and adolescents.

This regional perspective is the main contribution of the present study. Hartz et al. recently described nationwide trends in ADHD medication use in Norway from 2006 to 2022, including increasing use over time and changes in medication patterns (18). The present study extends this work by showing that the national increase did not occur uniformly across counties. Children and adolescents in different parts of Norway followed different dispensing trajectories over an 18-year period, despite living within the same universal healthcare system and national clinical framework (29). The findings therefore add a geographical treatment perspective to previous Norwegian register-based studies of ADHD medication use.

The observed regional variation should not be interpreted as direct evidence of either over-treatment in high-rate counties or under-treatment in low-rate counties. Dispensed medication is the endpoint of a clinical pathway that includes symptom recognition, help-seeking, referral, diagnostic assessment, treatment decisions, prescribing, family preferences, follow-up, and prescription redemption. Higher county rates may therefore reflect greater underlying need, better recognition, easier access to assessment, lower treatment thresholds, greater prescribing propensity, or differences in service organisation. Conversely, lower county rates may reflect lower underlying need, under-recognition, barriers to assessment, more restrictive treatment thresholds, or different local treatment practices. The present data cannot distinguish between these explanations.

The findings are consistent with previous Norwegian studies showing geographical variation in ADHD diagnosis and in clinician attitudes toward ADHD assessment and medication (23, 24, 30). However, the present study examined dispensed medication rather than diagnosis. This distinction is important. Diagnostic variation and medication-dispensing variation may overlap, but they are not identical. A county with higher diagnostic rates may not necessarily have proportionally higher medication-dispensing rates if treatment thresholds, parental preferences, availability of non-pharmacological interventions, or follow-up practices differ. Conversely, medication dispensing may vary because of prescribing practices even when diagnostic rates are similar. Future studies linking prescription data with diagnostic, clinical, service-level, and socioeconomic data are therefore needed to clarify which parts of the clinical pathway contribute most to regional differences.

The magnitude of the county differences is clinically and policy relevant. In 2022, dispensing prevalence among girls aged 6–11 years was more than three times higher in the highest-rate county than in the lowest-rate county. Among boys aged 12–17 years, the absolute difference between the highest and lowest county rates was 37.3 users per 1,000 inhabitants. Such differences are large enough to raise questions about equity of access, consistency of assessment practices, and regional variation in treatment thresholds. In a universal healthcare system, some regional variation is expected, but persistent two- to threefold differences suggest that local pathways and practice cultures may influence whether children and adolescents receive pharmacological ADHD treatment.

The geographical pattern observed in 2022 also suggests that regional variation was not random. Oslo was consistently among the counties with the lowest dispensing prevalence across sex and age strata, whereas higher adolescent rates were observed in several western, central, and northern counties, including Trøndelag, Rogaland, Vestland, Møre og Romsdal, and Nordland. These patterns may reflect differences in population composition, referral pathways, school and primary care recognition, specialist capacity, local clinical traditions, or attitudes toward ADHD medication. However, because the present study used aggregated prescription data, it cannot determine whether county differences were driven by population-level need, healthcare access, diagnostic practice, prescribing culture, or other contextual factors.

Sex differences should be interpreted within this regional framework. Boys had higher dispensing prevalence than girls in all counties and in both age groups, but the girl-to-boy rate ratio varied by county. This suggests that regional differences were not only differences in overall treatment level, but also differences in how boys and girls entered the medication-treatment pathway. The narrowing sex difference over time, particularly among adolescents, is consistent with improved recognition of ADHD in girls and with changes in clinical awareness and diagnostic practice (5–7, 31). However, these national sex-specific trends have already been described in previous work and should be regarded here mainly as context for interpreting regional differences.

The increase among adolescent girls in the latter part of the study period deserves particular caution. Increased recognition of inattentive and less externally disruptive presentations may have contributed to increased assessment and treatment among girls. Changes in diagnostic criteria may also have reduced barriers to diagnosis in adolescence, as DSM-5 increased the age-of-onset threshold and lowered the symptom-count requirement for older adolescents and adults (1). At the same time, the present study cannot determine whether the increase represents improved detection of previously under-recognised ADHD, changes in diagnostic thresholds, changes in help-seeking, or altered prescribing practices. Importantly, the regional analyses show that these changes were not uniform across counties.

The latter part of the study period overlapped with the COVID-19 pandemic. The pandemic may have influenced help-seeking, referral patterns, school functioning, family stress, and service access. However, the present design cannot distinguish pandemic-related effects from longer-term changes in awareness, diagnostic practice, service organisation, or prescribing. The pandemic period should therefore be regarded as a contextual factor rather than a causal explanation for the observed regional differences.

Medication substance patterns were included as secondary descriptive analyses. Methylphenidate-based treatment dominated throughout the study period, while the range of dispensed ADHD medications expanded over time, particularly after lisdexamfetamine and guanfacine became available in Norwegian clinical practice. These findings provide useful treatment context but are not the main contribution of the present study. The substance analyses were based on product-level outputs aggregated into active-substance categories and were non-mutually exclusive; the same individual could contribute to more than one active-substance category within a calendar year. They should therefore be interpreted as descriptive dispensing patterns by active substance, not as mutually exclusive patient-level treatment groups.

This study has several strengths. It used nationwide prescription register data covering an 18-year period, with complete capture of dispensed outpatient prescriptions in Norway (26, 27). Population denominators from Statistics Norway allowed calculation of annual county-, sex-, and age-specific dispensing prevalence (28). The harmonisation of historical county structures enabled comparison of regional trajectories over time. In addition, the study quantified regional variation using absolute rate differences and high-to-low county rate ratios, providing more interpretable measures of geographical variation than statistical significance testing alone.

Several limitations should be considered. NorPD records dispensed prescriptions, not ADHD diagnoses, symptom severity, clinical indication, adherence, medication ingestion, or treatment response. Dispensing prevalence is therefore a treatment measure and should not be interpreted as ADHD prevalence. First observed initiation in NorPD does not represent true lifetime treatment incidence, because ADHD medication use before 2005 and medication use outside Norway before immigration were not observable. The authors had access to aggregated outputs only and could not adjust for individual-level socioeconomic factors, comorbidity, diagnostic history, school factors, prescriber characteristics, or service availability. Some initiation outputs did not include all underlying deduplicated numerator counts by county, sex, and age group. Where approximate initiation counts were required for descriptive tabulation, they were derived from available rates or percentages and population denominators and should be interpreted as estimates. Inferential analyses were not based on these back-calculated counts. Product-level analyses were non-mutually exclusive, and cell counts below five were disclosure-protected in the supplied outputs.

The findings have clinical and policy relevance. Regional variation in dispensed ADHD medication should prompt further examination of whether children and adolescents across Norway have equitable access to assessment, evidence-based non-pharmacological interventions, medication initiation when appropriate, and systematic follow-up. The key question is not whether individual high-rate or low-rate counties are “right” or “wrong”, but whether regional differences reflect justified variation in need or unwarranted variation in clinical pathways and treatment thresholds. Future studies should link prescription data with diagnostic, clinical, educational, service-level, and socioeconomic information to clarify the drivers and consequences of these differences.

In conclusion, ADHD medication dispensing among Norwegian children and adolescents increased from 2005 to 2022, but the increase followed different county-level trajectories. In 2022, county-level dispensing prevalence differed by approximately two- to threefold across sex and age strata. This study extends previous Norwegian register-based work by showing substantial regional variation in pharmacological ADHD treatment within a universal healthcare system. Continued monitoring using transparent population denominators, together with studies linking prescription data to diagnostic, clinical, service-level, and socioeconomic information, is needed to determine whether these regional differences reflect variation in need, access, clinical practice, or treatment thresholds.

## Data Availability

The original contributions presented in the study are included in the article/**Supplementary Material**, further inquiries can be directed to the corresponding author/s.
