# Correction: County-level variation and trajectories in ADHD medication dispensing among Norwegian children and adolescents, 2005-2022: a nationwide prescription register study

**DOI:** 10.3389/fpsyt.2026.1989453

**Published:** 2026-09-16

**Authors:** Espen Anker, Bothild Bendiksen, Gro Janne Wergeland, Per Hove Thomsen

**Affiliations:** 1 Klinikk Toppetasjen, Oslo, Norway; 2 Department of Clinical Medicine, Faculty of Medicine, University of Bergen, Bergen, Norway; 3 Department of Clinical Medicine, Aarhus University, Aarhus, Denmark; 4 Aarhus University Hospital, Aarhus, Denmark

**Keywords:** ADHD, geographical variation, Norway, prescription trends, regional variation, sex differences, stimulant medication

The funder Medice Norway was erroneously omitted. Medice Norway supported registry access fees and made the statistical material available for this paper. Funders provided registry access only and had no role in design, analysis, or interpretation. The **Funding** statement has been updated.

The **Conflict of interest** statement was erroneously given as:

The author(s) declared that this work was conducted in the absence of any commercial or financial relationships that could be construed as a potential conflict of interest.

The correct statement is:

“Authors EA and BB were employed by the company Klinikk Toppetasjen. The authors GW and PT have received honoraria from Medice over the last three years.

The author(s) declared that this work received funding from Medice Norway. The funder had the following involvement in the study: supported registry access fees and made the statistical material available for this paper. The funder was not involved in the study design, analysis or interpretation.”

The original version of this article has been updated.

