# Supplementary material for: County-level variation and trajectories in ADHD medication dispensing among Norwegian children and adolescents, 2005-2022: a nationwide prescription register study
Source: Front Psychiatry. 2026 Sep 1;17:1861616. doi: 10.3389/fpsyt.2026.1861616 (PMC13574587; doi:10.3389/fpsyt.2026.1861616)
Supplement: Supplementary file 1 [file SupplementaryFile1.docx]

# Supplementary figures

Supplementary Figure S1. County-specific ADHD medication dispensing prevalence among girls aged 6-11 years, Norway 2005-2022. Each line represents one county. Rates are expressed as users per 1,000 inhabitants and were calculated using county-, sex-, age-, and year-specific population denominators from Statistics Norway. County groupings correspond to the 11-county structure used in the manuscript. The y-axis uses the same scale as in the other regional trend figures.

Supplementary Figure S2. County-specific ADHD medication dispensing prevalence among boys aged 6-11 years, Norway 2005-2022. Each line represents one county. Rates are expressed as users per 1,000 inhabitants and were calculated using county-, sex-, age-, and year-specific population denominators from Statistics Norway. County groupings correspond to the 11-county structure used in the manuscript. The y-axis uses the same scale as in the other regional trend figures.

Supplementary Figure S3. County-specific ADHD medication dispensing prevalence among girls aged 12-17 years, Norway 2005-2022. Each line represents one county. Rates are expressed as users per 1,000 inhabitants and were calculated using county-, sex-, age-, and year-specific population denominators from Statistics Norway. County groupings correspond to the 11-county structure used in the manuscript. The y-axis uses the same scale as in the other regional trend figures.

Supplementary Figure S4. County-specific ADHD medication dispensing prevalence among boys aged 12-17 years, Norway 2005-2022. Each line represents one county. Rates are expressed as users per 1,000 inhabitants and were calculated using county-, sex-, age-, and year-specific population denominators from Statistics Norway. County groupings correspond to the 11-county structure used in the manuscript. The y-axis uses the same scale as in the other regional trend figures.
